# Supplementary material for: Laser-Assisted Fabrication for Metal Halide Perovskite-2D Nanoconjugates: Control on the Nanocrystal Density and Morphology
Source: Nanomaterials (Basel). 2020 Apr 14;10(4):747. doi: 10.3390/nano10040747 (PMC7221537; doi:10.3390/nano10040747)
Supplement: Supplementary file 1 [file nanomaterials-10-00747-s001.pdf]

nanomaterials

Supporting Information

Laser-assisted fabrication for metal halide perovskite-2D nanocomposites: Control on the nanocrystal density and morphology

Athanasia Kostopoulou [1,*], Konstantinos Brintakis [1], Efthymis Serpetzoglou [1,2] and Emmanuel Stratakis [1,2,*]

[1] Institute of Electronic Structure and Laser, Foundation for Research and Technology—Hellas, Heraklion, 71110 Crete, Greece; kbrin@iesl.forth.gr (K.B.); eserpe@iesl.forth.gr (Ef.S.)
[2] Department of Physics, University of Crete, 71003 Heraklion, Crete, Greece
* Correspondence: akosto@iesl.forth.gr (A.K.); stratak@iesl.forth.gr (E.S.); Tel.: +30-2810-391874 (A.K.); Tel.: +30-2810-391274 (E.S.)

Received: 12 March 2020; Accepted: 8 April 2020; Published: date

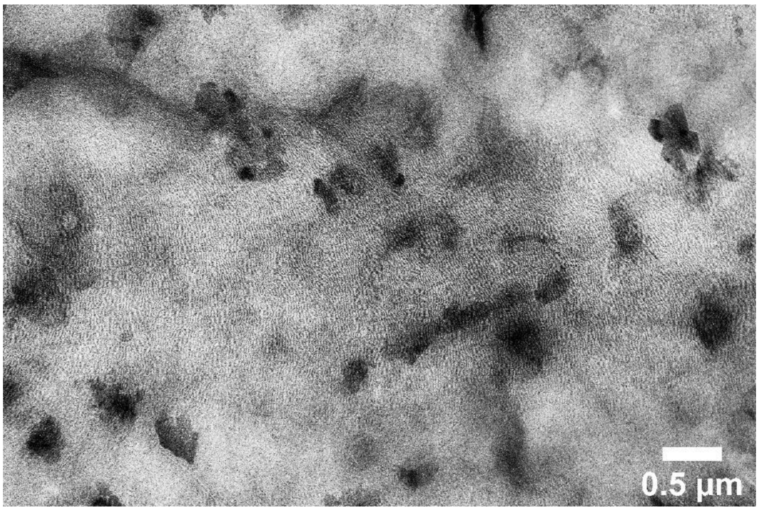

Figure S1.TEM image of the irradiated solution of GO with metal halide nanocrystals. The GO was exfoliated in DMF while the nanocrystals are dispersed in toluene. The nanocrystals are destroyed.

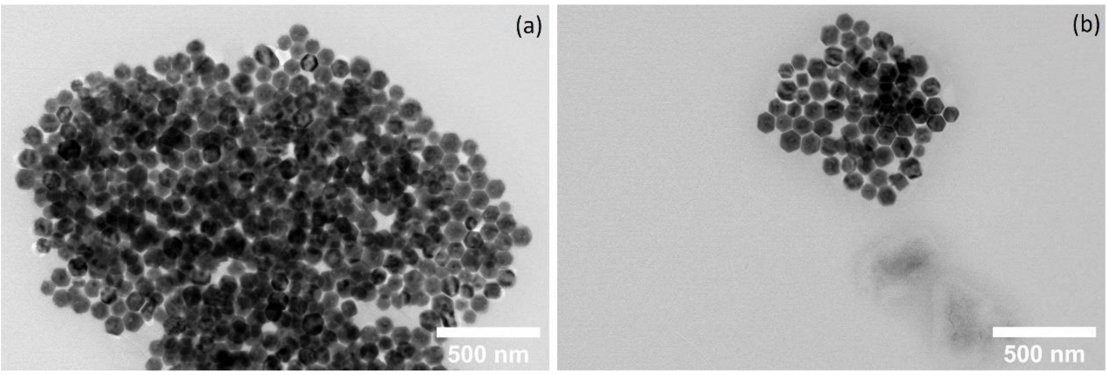

Figure S2. (**a-b**) TEM images of the mixture of the nanocrystals and the GO before the laser irradiation.

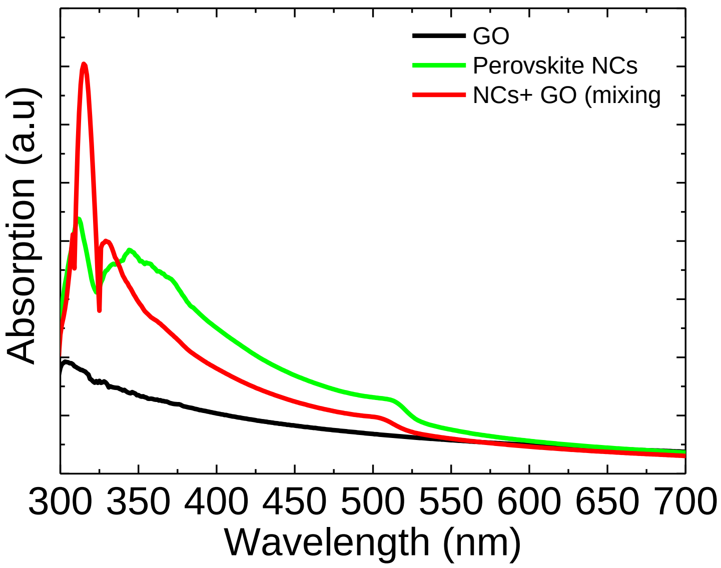

Figure S3. Absorption spectra of the metal halide nanocrystals (green curve), the exfoliated GO (black curve) and the mixture of the two (red curve) before the laser irradiation in DCB.

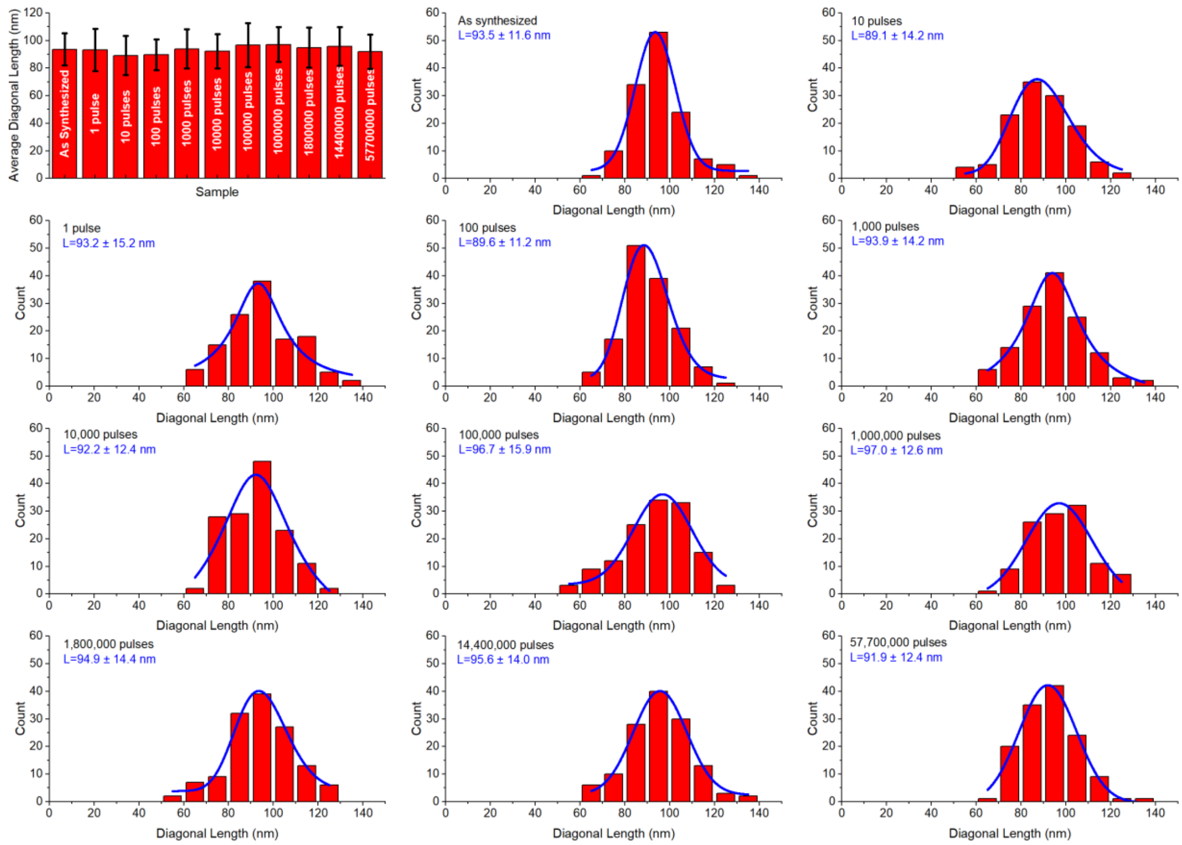

Figure S4. Size distribution diagrams for the nanohexagons before and after the laser irradiation. The diagonal length (L) was extracted from the statistics of at least 130 hexagonal nanocrystals in each sample. A very small fluctuation of the average diagonal length upon irradiation is observed which lays within the error range.

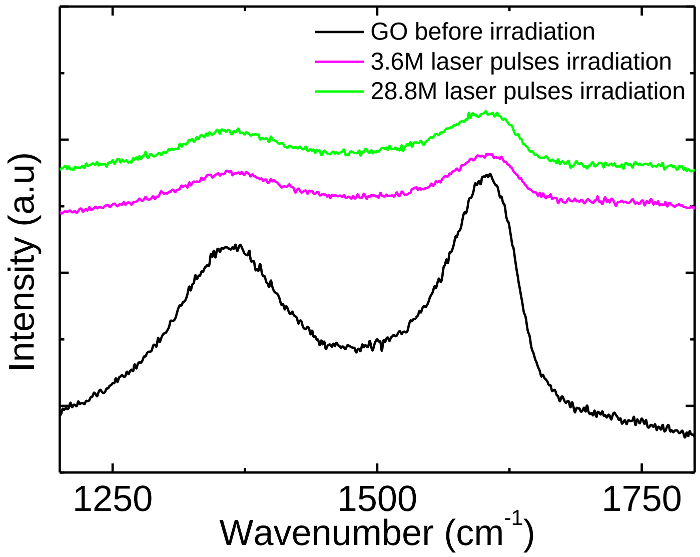

Figure S5. Raman spectra of the GO used for the fabrication of the hybrid system before (black line) and after 3.6 and 28.8 million pulses laser irradiation (green and purple curves).

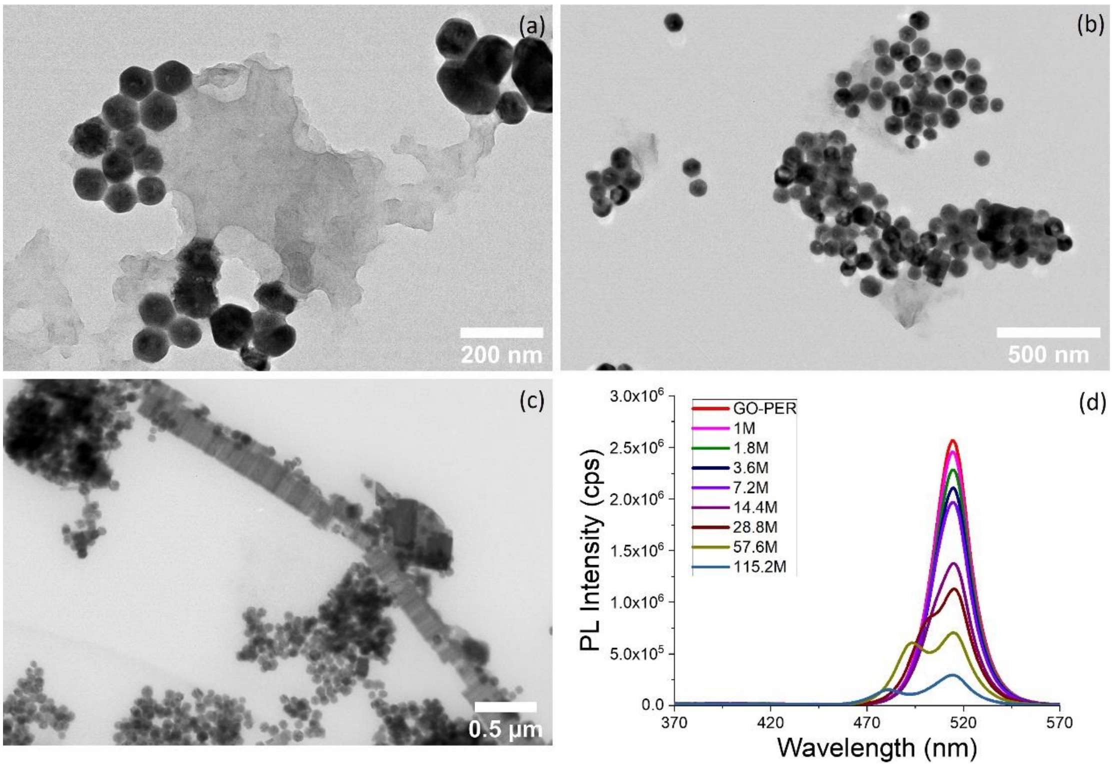

Figure S6. TEM images and PL spectra for irradiation duration longer than 1 × 10^6 pulses.

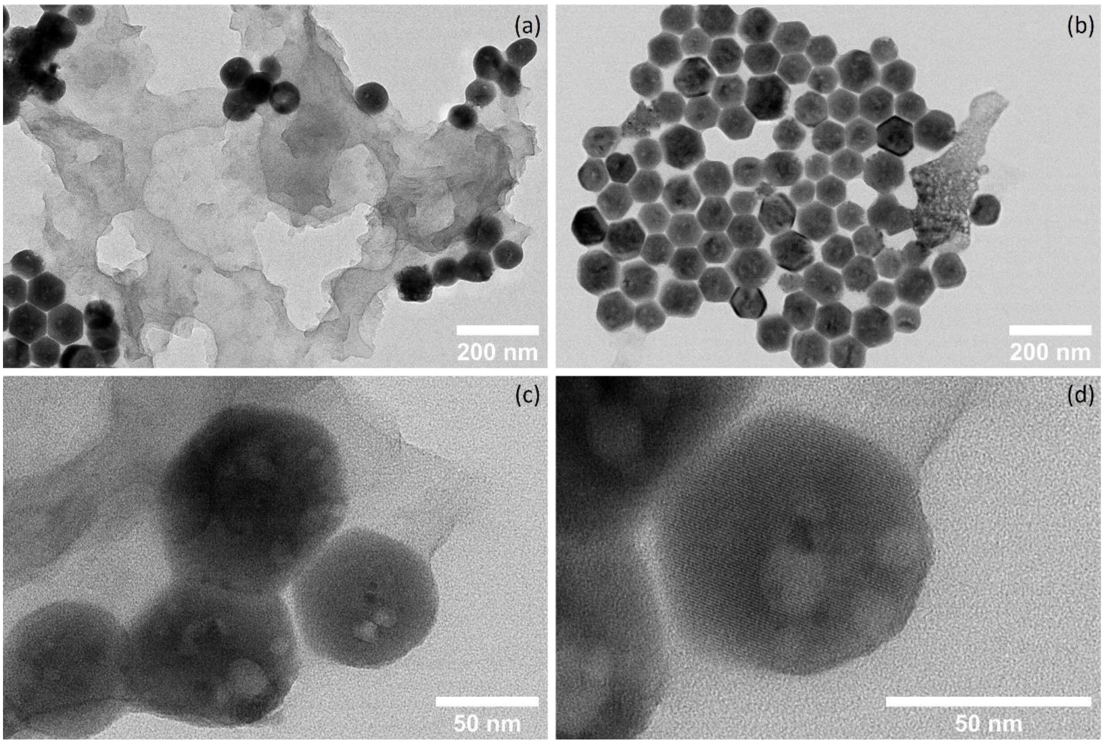

Figure S7. TEM images of the GO-perovskite composite after 10^6 pulses irradiation.

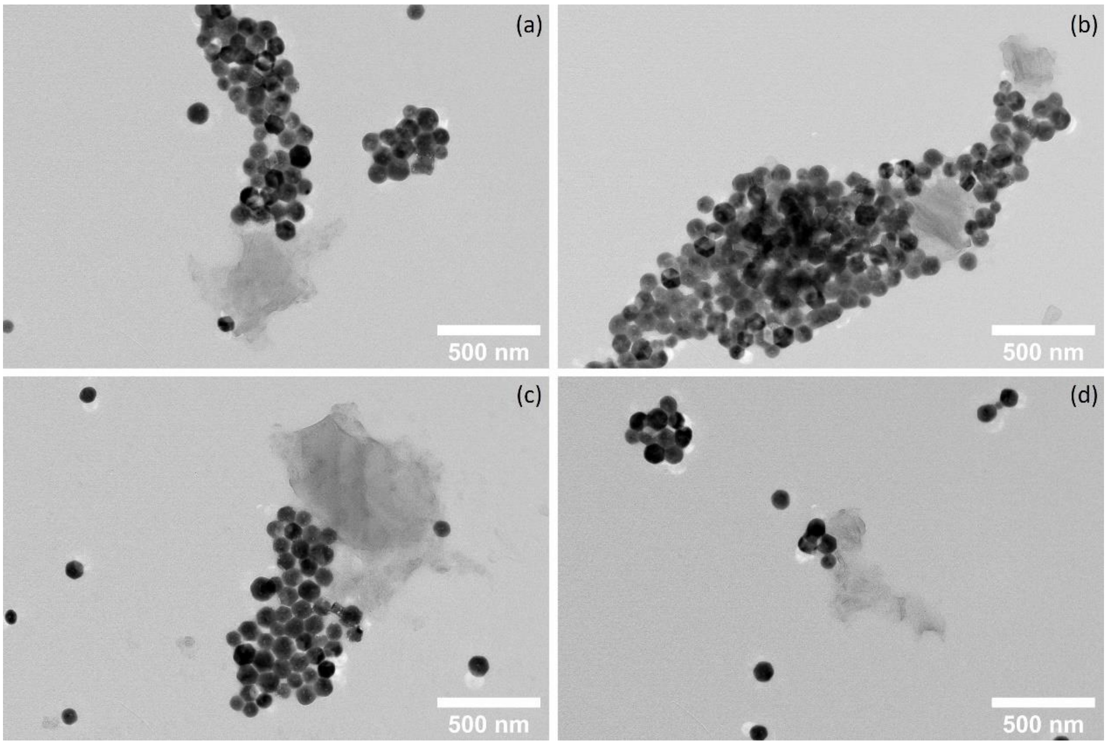

Figure S8. TEM images of the GO-perovskite composite after 1.8×10^6 pulses irradiation.

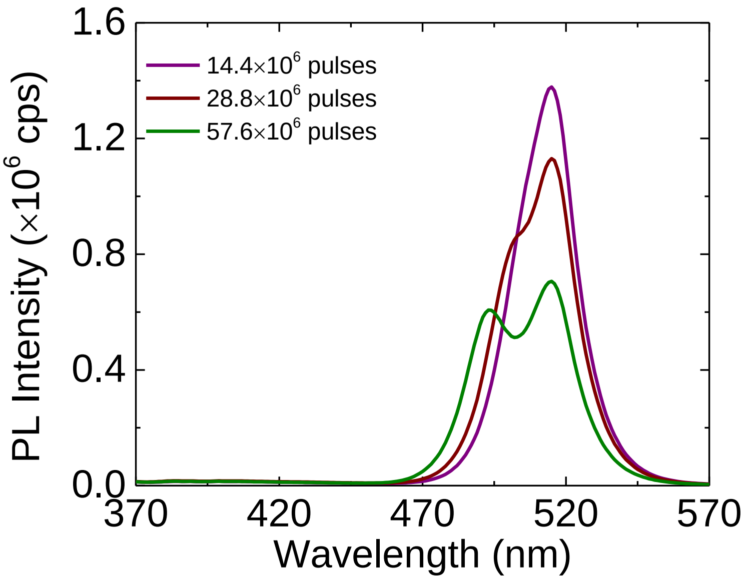

Figure S9. PL spectra of the perovskite-GO composite solution after irradiation with 14.4, 28.8 and 57.6 × 10^6 pulses.

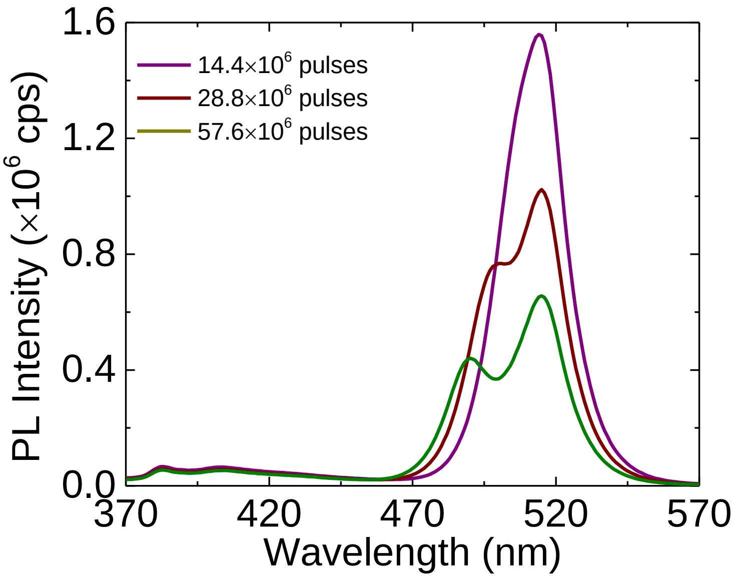

Figure S10. PL spectra of the as-prepared nanohexagons solution after irradiation with 14.4, 28.8 and 57.6 × 10^6 pulses.

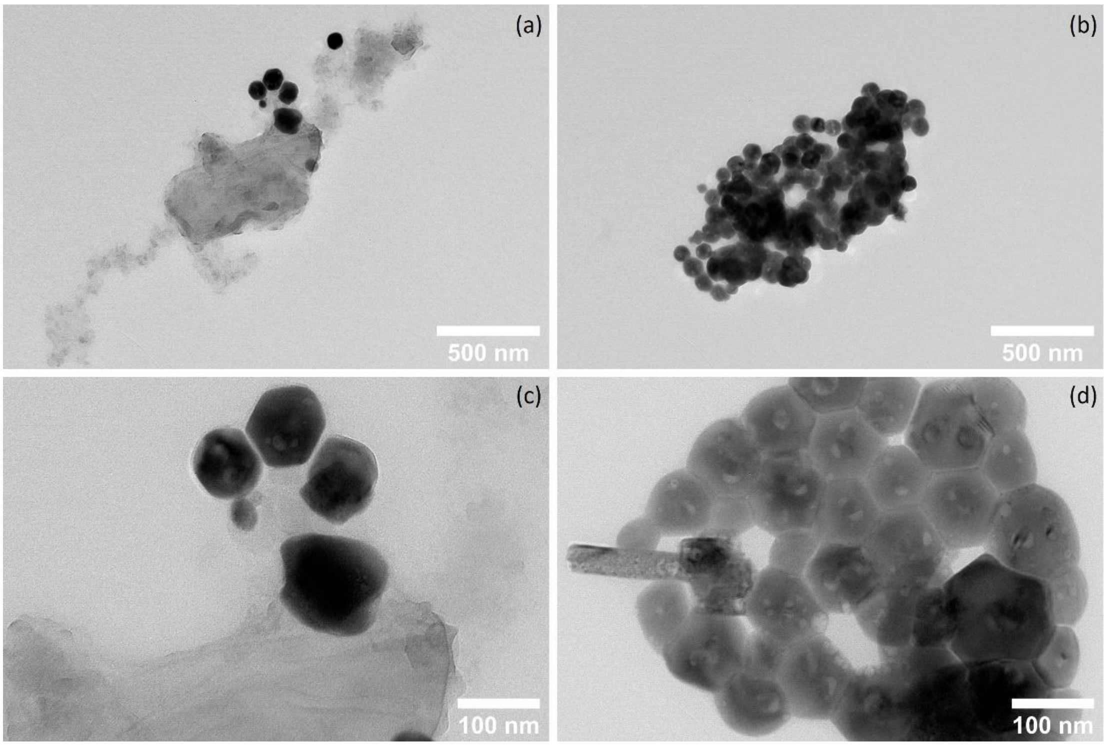

Figure S11. TEM images of the GO-perovskite composite after 14.4 × 10^6 pulses irradiation.

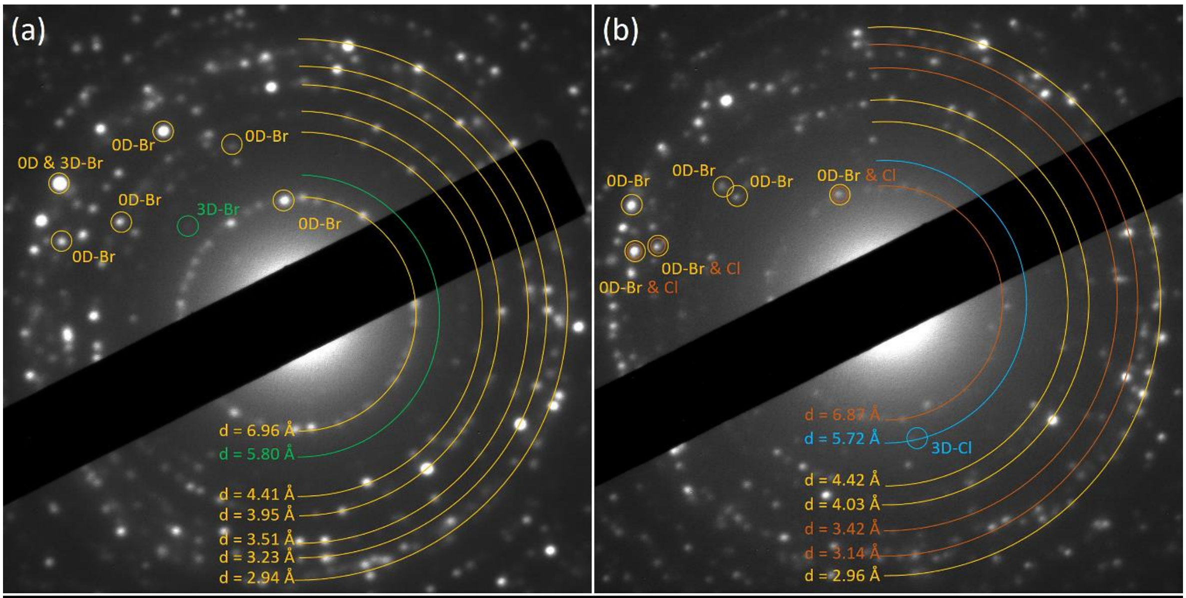

Cs_4PbBr_6 (0D-Br)	Intensity (Norm 100)	$CsPbBr_3$ (3D-Br)	Intensity (Norm 100)	Cs_4PbCl_6 (0D-Cl)	Intensity (Norm 100)	$CsPbCl_3$ (3D-Br)	Intensity (Norm 100)
ICSD: 25124		*ICSD: 97851*		*ICSD: 35703*		*mp-675524*	
$d_{(012)}$=7.00 Å	82.5	$d_{(101)}$=6.70 Å	1.9	$d_{(012)}$=6.72 Å	31.1		
		$d_{(002)}$=5.88 Å	29.1			$d_{(002)}$=5.72 Å	13.1
$d_{(113)}$=4.41 Å	44.1	$d_{(112)}$=4.14 Å	45.8	$d_{(113)}$=4.24 Å	51.8		
$d_{(300)}$=3.96 Å	75.6	$d_{(200)}$=4.10 Å	49.7	$d_{(300)}$=3.81 Å	100	$d_{(102)}$=4.06 Å	93.7
$d_{(024)}$=3.50 Å	50.6	$d_{(103)}$=3.54 Å	16.7	$d_{(024)}$=3.36 Å	41.6	$d_{(122)}$=3.31 Å	1.0
$d_{(131)}$=3.24 Å	26.3	$d_{(113)}$=3.25 Å	8.4	$d_{(131)}$=3.11 Å	24.3	$d_{(131)}$=3.06 Å	1.4
$d_{(223)}$=2.94 Å	100	$d_{(004)}$=2.94 Å	100	$d_{(223)}$=2.83 Å	49.7	$d_{(200)}$=2.88 Å	45.5

Figure S12. Selected area (electron) diffraction patterns of the as prepared nanocrystals (a) and the 14.4×10^6 pulses irradiated samples (b), respectively. The measured spots are circled with the analogous color corresponding to the phases referred on the table below. Colored semi-rings of the measured *d*-spacing are drawn to indicate the measured distance and the corresponding phase. It has to be noted that the 3D phases ($CsPbBr_3$ and $CsPbCl_3$) have very low intensity peaks in the diffraction patterns or are masked by the 0D counterparts. The table comprises by the correlated reference peaks of the pdf cards and their intensities are normalized to 100.

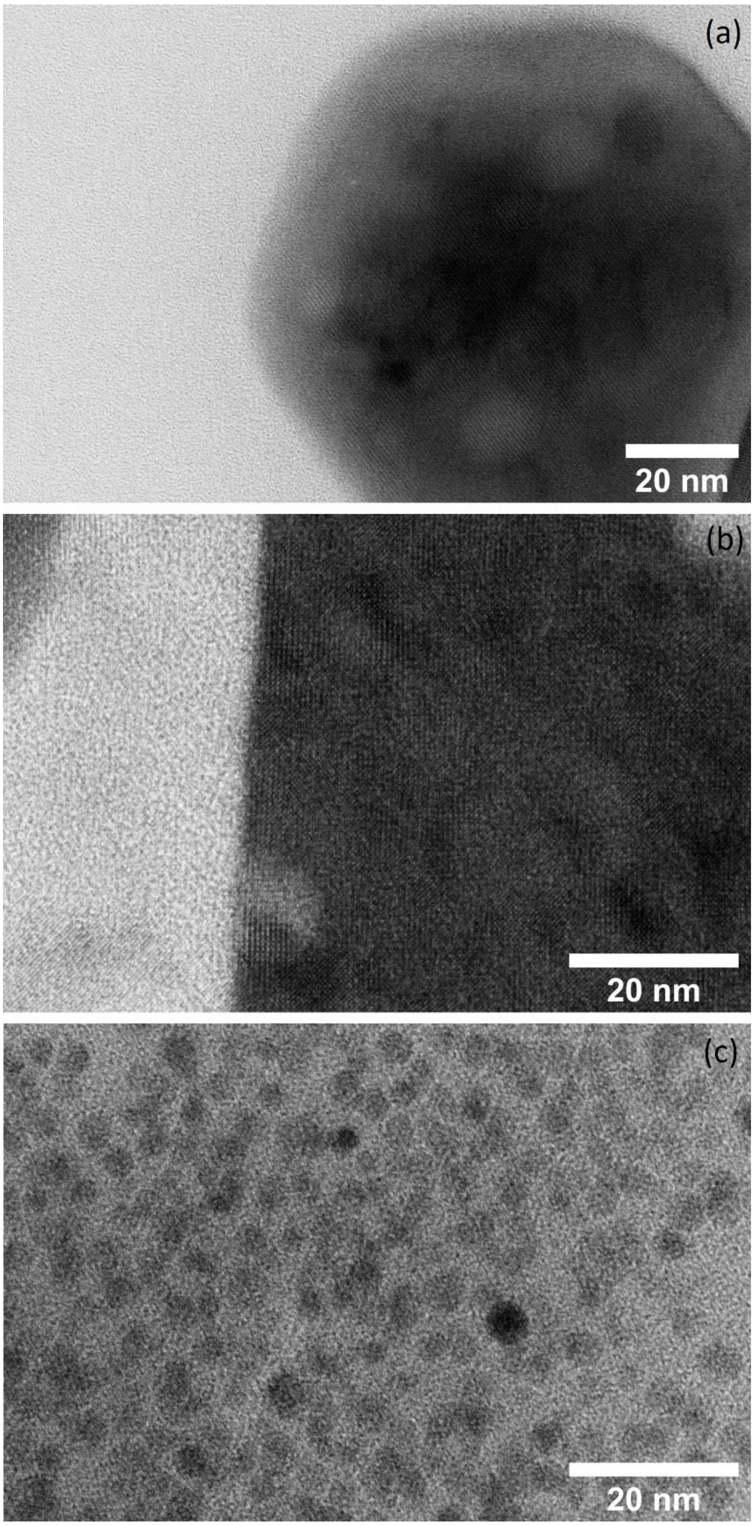

Figure S13. Different nanocrystal morphologies such as nanohexagons (a), nanoribbons (b) and quite spherical observed in the solution after irradiation with 57.6x10^{6} pulses.

© 2020 by the authors. Submitted for possible open access publication under the terms and conditions of the Creative Commons Attribution (CC BY) license (http://creativecommons.org/licenses/by/4.0/).
